# Identification of the First ATRIP–Deficient Patient and Novel Mutations in ATR Define a Clinical Spectrum for ATR–ATRIP Seckel Syndrome

**DOI:** 10.1371/journal.pgen.1002945

**Published:** 2012-11-08

**Authors:** Tomoo Ogi, Sarah Walker, Tom Stiff, Emma Hobson, Siripan Limsirichaikul, Gillian Carpenter, Katrina Prescott, Mohnish Suri, Philip J. Byrd, Michiko Matsuse, Norisato Mitsutake, Yuka Nakazawa, Pradeep Vasudevan, Margaret Barrow, Grant S. Stewart, A. Malcolm R. Taylor, Mark O'Driscoll, Penny A. Jeggo

**Affiliations:** 1 Nagasaki University Research Centre for Genomic Instability and Carcinogenesis (NRGIC), Nagasaki University, Sakamoto, Nagasaki, Japan; 2 Department of Molecular Medicine, Atomic Bomb Disease Institute, Nagasaki University, Sakamoto, Nagasaki, Japan; 3 Double Strand Break Repair Laboratory, Genome Damage and Stability Centre, University of Sussex, Brighton, United Kingdom; 4 Department of Clinical Genetics, Chapel Allerton Hospital, Leeds, United Kingdom; 5 Human DNA Damage Response Disorders Group, Genome Damage and Stability Centre, University of Sussex, Brighton, United Kingdom; 6 Clinical Genetic Service, City Hospital, Nottingham, United Kingdom; 7 School of Cancer Sciences, College of Medical and Dental Sciences, University of Birmingham, Birmingham, United Kingdom; 8 University Hospitals of Leicester NHS Trust, Leicester Royal Infirmary, Leicester, United Kingdom; University of Oxford, United Kingdom

## Abstract

A homozygous mutational change in the *Ataxia-Telangiectasia and RAD3 related* (*ATR*) gene was previously reported in two related families displaying Seckel Syndrome (SS). Here, we provide the first identification of a Seckel Syndrome patient with mutations in *ATRIP*, the gene encoding ATR–Interacting Protein (ATRIP), the partner protein of ATR required for ATR stability and recruitment to the site of DNA damage. The patient has compound heterozygous mutations in *ATRIP* resulting in reduced ATRIP and ATR expression. A nonsense mutational change in one *ATRIP* allele results in a C-terminal truncated protein, which impairs ATR–ATRIP interaction; the other allele is abnormally spliced. We additionally describe two further unrelated patients native to the UK with the same novel, heterozygous mutations in *ATR*, which cause dramatically reduced ATR expression. All patient-derived cells showed defective DNA damage responses that can be attributed to impaired ATR–ATRIP function. Seckel Syndrome is characterised by microcephaly and growth delay, features also displayed by several related disorders including Majewski (microcephalic) osteodysplastic primordial dwarfism (MOPD) type II and Meier-Gorlin Syndrome (MGS). The identification of an ATRIP–deficient patient provides a novel genetic defect for Seckel Syndrome. Coupled with the identification of further ATR–deficient patients, our findings allow a spectrum of clinical features that can be ascribed to the ATR–ATRIP deficient sub-class of Seckel Syndrome. ATR–ATRIP patients are characterised by extremely severe microcephaly and growth delay, microtia (small ears), micrognathia (small and receding chin), and dental crowding. While aberrant bone development was mild in the original ATR–SS patient, some of the patients described here display skeletal abnormalities including, in one patient, small patellae, a feature characteristically observed in Meier-Gorlin Syndrome. Collectively, our analysis exposes an overlapping clinical manifestation between the disorders but allows an expanded spectrum of clinical features for ATR–ATRIP Seckel Syndrome to be defined.

## Author Summary

Seckel Syndrome (SS) is a rare human disorder characterised by small head circumference and delayed growth. Patients can show additional features including abnormal bone development, receding chins, sloping foreheads, and small ears. In 2003, we identified *ataxia telangiectasia and Rad3 related* (*ATR*) as a causal genetic defect in two related families displaying SS. However, additional patients with mutations in *ATR* have not hitherto been identified. Here, we describe two further patients with novel mutations in *ATR*. Additionally, we identify a patient with mutations in *ATRIP*, which encodes an interacting partner of ATR, representing a novel genetic defect causing SS. ATR functions to promote the ability of cells to recover from difficulties encountered during replication. We show that patient-derived cells have reduced ATR and ATRIP protein levels and defective ATR/ATRIP function. Our identification of further ATR–ATRIP defective patients and a consideration of their clinical features aids the characterisation and identification of this form of SS and provides insight into the role played by the ATR–ATRIP complex during development.

## Introduction

Seckel Syndrome (SS) (OMIM 216000) is an autosomal recessive disorder characterised by marked microcephaly, intra-uterine and post-natal growth retardation, developmental delay and characteristic facial features, encompassing micrognathia (small and receding chin), receding forehead and pronounced nose [1]. Majewski (microcephalic) osteodysplastic primordial dwarfism (MOPD) type II and Meier-Gorlin Syndrome (MGS) also display microcephaly and primordial dwarfism [2], [3]. However, each of these disorders display an additional spectrum of features conferring clinical distinction. Despite this, on an individual basis, assigning patients to a specific classification is difficult. Additionally, primary microcephaly represents a disorder displaying pronounced microcephaly without marked impact on growth [4]. Five loci conferring SS have been described with four genes identified [5], [6]. The first causal genetic defect identified for SS was the *Ataxia-Telangiectasia and RAD3 related (ATR)* gene [7]. A homozygous mutation in *ATR* was identified in two related SS families and cell-based studies provided strong evidence for an impact on ATR function in patient cell lines. This sub-class of SS was designated ATR–SS. More recently, mutations in *CTIP* were identified in a SS patient as well as in a family described as displaying Jawad Syndrome [8]. Additionally, mutations in CENPJ and CEP152, two centrosomal proteins, have been described in SS patients, although mutations in these genes more frequently confer primary microcephaly [9], [10]. Mutations in *PCNT*, which encodes a centrosomal protein, and *ORC1L*, a component of the original licensing complex, were reported in patients originally classified as SS although in both cases retrospective analysis revealed that such mutations more frequently cause MOPD type II or MGS, respectively, highlighting the diagnostic challenge faced in the clinic [11]–[15]. These studies demonstrate that evaluation of multiple patients is required to provide insight into the spectrum of clinical features conferred by specific gene defects, which ultimately aids an understanding of the role of the defective protein during development. To date all ATR–SS patients belong to one of two related families, which harbour the identical homozygous mutation in *ATR*, thereby limiting the characterisation of the clinical phenotype conferred by ATR deficiency. Furthermore, no patients deficient in ATR interacting protein, ATRIP, which is required for ATR stability, have hitherto been described.

ATR, like the related Ataxia-Telangiectasia mutated (ATM) protein, is a phosphoinositol-3 kinase (PI3)-like kinase that functions at the centre of a signal transduction network activated by DNA damage, and most importantly, by replication fork stalling [16]. ATR and ATM share phosphorylation targets but whilst ATM is activated by DNA double strand breaks (DSBs) that arise, for example, following exposure to ionising radiation (IR), ATR is activated by single stranded (ss) regions of DNA that arise following replication fork stalling or exposure to agents that induce bulky DNA adducts [17], [18]. Importantly, since replication fork stalling occurs during most cycles of replication, ATR is essential. ATM, in contrast, is non-essential presumably because endogenous DSBs arise infrequently. ATR forms a stable complex with ATR–interacting protein (ATRIP), which is required for ATR stability [19]. Further, ATRIP is required for ATR localisation to ssDNA regions and hence for ATR activation. Consequently, in a range of organisms loss of ATRIP or its homologue, phenocopies ATR deficiency [17], [20]–[22]. Although ATM and ATR share overlapping substrates, ATR specifically phosphorylates Chk1 whilst ATM phosphorylates the related kinase, Chk2. The major functions of ATR are to activate cell cycle checkpoint arrest, stabilise stalled replication forks and promote replication fork restart, which is achieved through its ability to phosphorylate a range of substrates including p53 and H2AX [18], [23], [24]. Interestingly, in the context of SS, CtIP promotes DNA end resection, which leads to ss DNA formation, the lesion activating ATR. Hence, CtIP functions in a mechanism leading to ATR activation. It is noteworthy that cells derived from *PCNT*-mutated MOPD type II patients are also defective in ATR–dependent G2/M checkpoint arrest although upstream steps in the ATR–signalling pathway are activated normally [11]. These findings suggest that PCNT is required for an important end-point of ATR function. Additionally, the origin licensing complex, components of which are mutated in MGS, is required for the initiation of replication and *ORC1L*-deficient MGS cell lines display slow S phase progression [13]. Similarly, ATR promotes S phase progression by facilitating recovery from replication fork stalling. Together, these findings demonstrate mechanistic overlap between ATR, PCNT and ORC1L, which may underlie some clinical overlap in the disorders conferred by mutations in the genes encoding these proteins.

Here, we provide the first description of a SS patient with mutations in *ATRIP*. Interestingly, the mutational change in one *ATRIP* allele causes impaired ATR–ATRIP interaction and our extensive cellular analysis confirms a deficiency in ATR signalling and damage responses. Additionally, we describe two further, unrelated patients with mutations in *ATR*. The identification and clinical description of an ATRIP patient and two further ATR patients provides a more definitive characterisation of the clinical phenotype conferred by ATR deficiency.

## Results

### Cells derived from patient CV1720 display a compromised DNA damage response

**Figure 1:**
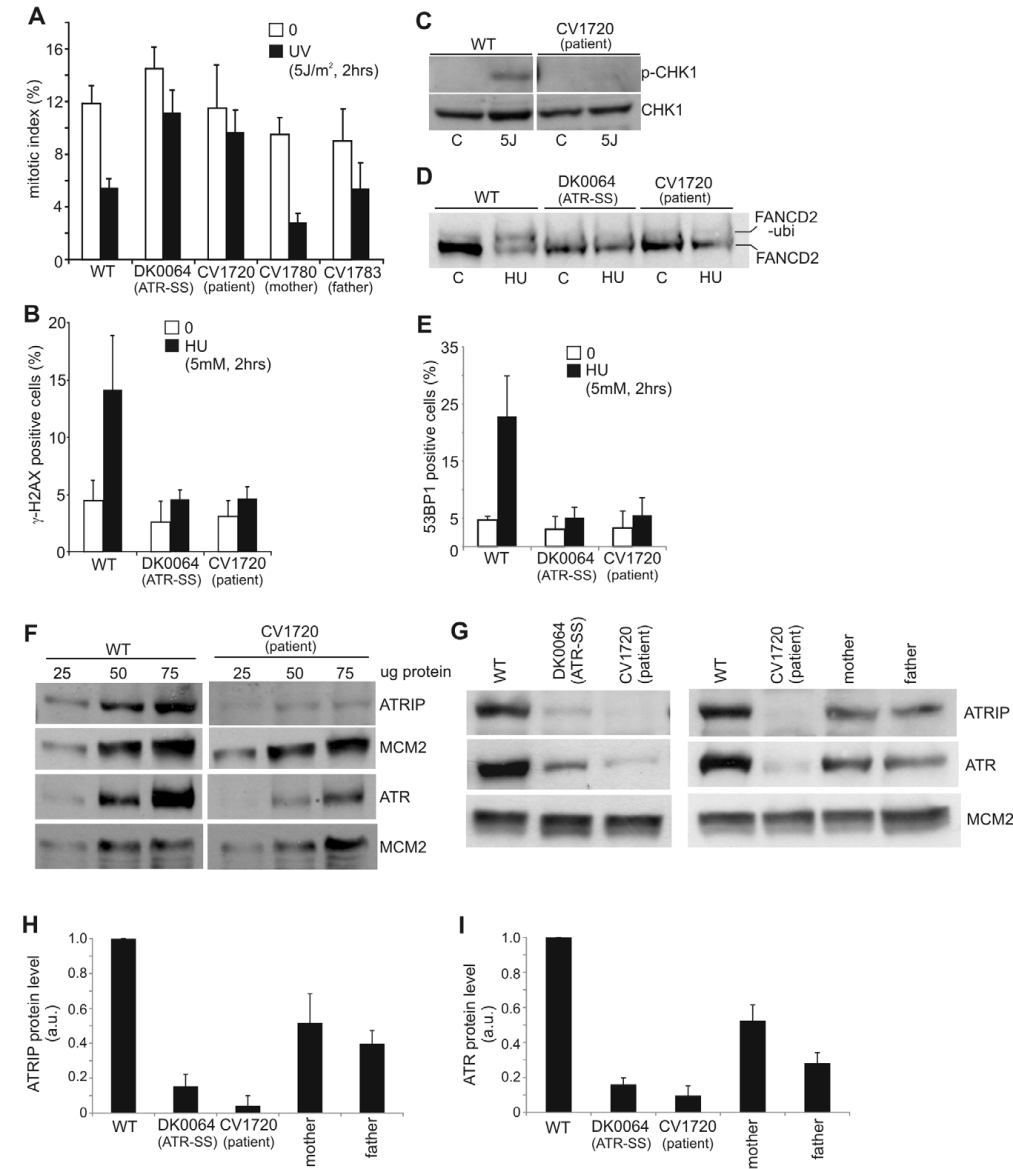
CV1720 cells show impaired ATR–dependent DNA damage responses. A) WT, DK0064 (ATR–SS), CV1720 (patient), CV1780 (patient's mother) and CV1783 (patient's father) cells were exposed to 5 Jm^−2^ UV and the mitotic index (MI) assessed 2 h post exposure. A greater than two fold decrease in mitotic index is observed in WT and both paternal cell lines but not in DK0064 (ATR–SS) or CV1720 (patient) cells. B) Cells were exposed to 5 mM HU for 2 h and the percentage of p-H2AX (γ-H2AX) positive cells assessed by immunofluorescence. Note that HU causes pan nuclear p-H2AX formation rather than defined foci as observed after exposure to ionising radiation. Thus, the percentage of γ-H2AX positive cells was scored. C) Cells were exposed to UV (5 Jm^−2^) and subjected to Western Blotting (WB) using p-Chk1 (p-Ser317) antibodies at 2 h. Chk1 expression was shown to be similar in WT and patient cells (lower panel). D) Cells were exposed to 3 mM HU for 2 h and whole cell extracts analysed by WB using FANCD2 antibodies. The ubiquitylation of FANCD2, detectable by a product with reduced mobility, is diminished in DK0064 (ATR–SS) and CV1720 cells compared to WT cells. E) Cells were exposed to 5 mM HU and examined for the percentage of cells showing >5 53BP1 foci at 2 h post exposure. 53BP1 foci formation is reduced in DK0064 (ATR–SS) and CV1720 cells compared to WT cells. F–I) The indicated cells were processed by WB using ATRIP or ATR antibodies. MCM2 was used as a loading control. F shows the analysis of a range of protein levels for accurate comparison. CV1720 (patient) cells show markedly reduced ATR and ATRIP protein levels. G shows that both parental lines have approximately half the level of ATR and ATRIP compared to two WT cell lines. DK0064 (ATR–SS) and CV1720 cells, in contrast, have more dramatically reduced ATR and ATRIP protein levels. 50 ug protein was loaded. WT in all panels was GM2188. Patient, mother and father were as shown in panel A. H and I show the quantification of ATRIP and ATR protein levels from at least three independent WB experiments.

Patient CV1720 displayed severe microcephaly, growth delay and dysmorphic facial features and was classified as a SS patient (see Table 1 and Figure S1A for details of the clinical features). Cell line CV1720 is a lymphoblastoid cell line (LBL) derived from the patient; fibroblasts were not available. Cells from the previously described ATR–SS (DK0064) patient display impaired DNA damage responses and phosphorylation of ATR substrates [7]. To determine whether CV1720 cells are defective in ATR–dependent G2/M checkpoint arrest, the mitotic index (MI) was monitored at 2 h following UV exposure, a form of DNA damage known to activate ATR–dependent checkpoint arrest. Whilst WT LBLs show a significantly reduced MI following UV exposure compared to undamaged cells, CV1720 cells showed only a modest decrease similar to that observed in DK0064 (ATR–SS) cells (Figure 1A). Cells from the parents of patient CV1720 (CV1780 and CV1783) displayed normal G2/M checkpoint arrest.

**Table 1:** Clinical features of ATR/ATRIP–deficient patients.

	ATRIP–SS		ATR–SS	27-4BI	19-8BI
Ethnicity	Gujarati-Indian (consanguineous)		Pakistani (consanguineous)	English	English
**Birth.**					
**OFC (cm)**	27.1		24 *(-8SD)*	27	24.2
**Wgt (Kg)**	2.06		1.1 *(-3SD)*	1.15	0.77
**Hgt (cm)**	NR		NR	36	NR
**Age.**	**14 mts**	**3 yrs 3 mts**	**9 yrs**	**20 mts**	**4.5 yrs**
**OFC (cm)**	-9SD	-10SD	-12SD	-10SD	-10SD
**Wgt (Kg)**	-5SD	-6SD	-3.3SD	-8SD	-7SD
**Hgt (cm)**	-5SD	-6.5SD	NR	-8SD	-8SD
**Face**	Micrognathia, receding forehead, prominent nose.		Micrognathia, receding forehead, prominent nose.	Micrognathia, prominent nose, hypoplastic alae nasi, low set columella, deep set short palpebral fissures.	Micrognathia, blepharophimosis, short palpebral fissures. Prominent nose; high nasal bridge. High anterior hairline.
**Teeth**	Dental crowding.		Dental crowding and malocclution.	4 teeth at 20 months.	Dental crowding.
**Ears**	Small lobes.		Posteriorly rotated with absent lobes.	Small, round, low set with poorly formed antihelix tragus & antitragus. Absent lobes.	Small ears with absent lobes
**Hands**	Bilateral 5^th^ finger clinodactyly.		Multiple ivory epiphysis.	Small, tapering fingers.	Bilateral 5^th^ finger clinodactyly. 5^th^ metacarpels appear short. Blue colouration to both thenar eminence.
**Skeletal Survey**	Delayed bone age (wrist & hips), symmetric dwarfism.		Microcrania with fuse sutures. Mild thoracic kyphosis. Ribs angulated posteriorly. Narrow iliac blades, cox valga and minor subluxation of the hips. No disslocation of the radial heads	Symmetric dwarfism. Small patellae. No joint hypermobility or kyphoscoliosis.	Symmetric dwarfism. Copper beaten skull. No ossification of the patellae (age 4 yrs). Marked hip & shoulder flexibility. No kyphosis.
**Endo-crinology**	Normal IGF1, TFT, LH, FSH & cortisol.		NA	NA	NA
**MRI**	14 mts:generalised cerebral atrophy, normal ventricular systems. Delayed myelination in the anterior limb of the internal capsule. Pituitary is present though of unusual shape with absent fossa.		NA	NA	2 yrs: abnormal gyration in posterior aspect of the cingulated gyrus extending into the parietal occipital region. Hypoplastic corpus collasum.
**Other**	NR		Developmental delay. Walked at 7 yrs.	No abnormal skin pigmentation. Small feet with metatarsus adductus	No abnormal skin pigmentation.Developmental delay. Sat at 15 mts, walked at 3 yrs 10 mts. High pitched voice, asthma, multiple chest infections, feeding difficulties-reflux (gastrostomy fed). Multiple liver cysts consistent with Caroli's disease found at 17 mts.

NR; not recorded. NA; not assessed.

We have previously observed that cells from other SS patients display defects in ATR–dependent G2/M checkpoint arrest but activate upstream steps in the ATR signalling cascade normally [25]. This is exemplified by cell lines from MOPD type II patients with mutations in *PCNT*, which are defective in ATR–dependent G2/M checkpoint arrest but proficient in ATR phosphorylation events [11]. Therefore, next, we examined whether CV1720 LBLs efficiently activate upstream steps in ATR signalling. Since these assays predominantly reflect the response of replicating phase cells, we first verified that CV1720 and control LBLs harbour a similar percentage of S phase cells (Figure S2). Pan-nuclear phosphorylation of H2AX (γH2AX) after replication fork stalling represents an ATR–specific damage response [24]. Strikingly, whilst exposure to 5 mM HU for 2 h resulted in an elevated number of cells staining positively for γH2AX in WT cells, this was not observed in either CV1720 or DK0064 (ATR–SS) cells (Figure 1B). We note that although previous studies have shown that ATM can be activated and phosphorylate γH2AX at DSBs arising following HU treatment in the absence of ATR due to enhanced fork collapse, this was not observed at 2 h post 5 mM HU exposure in these patient cells most likely due to residual ATR activity and/or the early times examined [26], [27]. Chk1 represents an important ATR substrate required for G2/M checkpoint arrest. To examine Chk1 activation, we carried out Western Blotting using p-Chk1 antibodies. Following the same UV exposure conditions (2 h post 5 Jm^−2^) employed to examine G2/M checkpoint arrest, we observed a pronounced p-Chk1 band in WT LBLs but not in CV1720 cells although Chk1 levels were similar in the two lines (Figure 1C). These results provide strong evidence that CV1720 show impaired ATR–dependent substrate phosphorylation.

A further ATR–dependent response is mono-ubiquitylation of FANCD2 following exposure to HU [28]. Mono-ubiquitinated FANCD2 can be detected by the presence of a slowly migrating isoform of FANCD2 generated post exposure to 3 mM HU. Whilst such a product was detected in WT cell extracts, it was absent in CV1720 and DK0064 (ATR–SS) cell extracts (Figure 1D). Finally, ATR also regulates the formation of 53BP1 foci following replication fork stalling via a Chk1-dependent process. We observed a failure to form 53BP1 foci following exposure to 5 mM HU in CV1720 and DK0064 (ATR–SS) LBLs in contrast to WT LBLs (Figure 1E), consistent with the diminished levels of p-Chk1 observed in CV1720 cells.

Collectively, these studies provide strong evidence that CV1720 cells are defective in an upstream step of the ATR–dependent signalling response defining them as distinct to the majority of previously characterised SS cell lines, which, though defective in UV-induced G2/M checkpoint arrest, are proficient in upstream steps of the ATR signalling response [25].

### Reduced ATR and ATRIP protein expression in CV1720 cells

Given the overlapping cellular phenotype between CV1720 and DK0064 (ATR–SS) cells, we examined CV1720 cells for expression of ATR and ATRIP protein by Western Blotting. Strikingly, we observed markedly reduced levels of both ATR and ATRIP in CV1720 cells (Figure 1F). Since ATRIP stabilises ATR, this does not distinguish whether the primary defect lies in ATR or ATRIP and indeed a similar reduced level of ATR and ATRIP was observed in DK0064 (ATR–SS) cells (Figure 1G). Significantly, we observed reduced ATRIP and ATR in both parental LBLs (CV1780 and CV1783), which was approximately 50% of the level in WT LBLs (Figure 1G–1I).

### Sequencing analysis reveals mutational changes in *ATRIP* in CV1720 cells

To examine whether the causal genetic defect in CV1720 lies in *ATR* or *ATRIP*, we carried out sequencing of the two genes. First, we undertook PCR-based gDNA sequencing of the 47 exons and neighbouring exon-intron boundaries of the human *ATR* gene from CV1720 cells and failed to observe any mutational changes likely to be of functional significance. Next, we undertook gDNA sequencing of *ATRIP* exons and observed a heterozygous mutational change, c.2278C>T, in exon12 which generated a stop codon predicting a truncated protein at position arginine 760 (p.Arg760*) (Figure S3). However, no mutational changes in any other exons were identified although we detected several novel intronic changes that could potentially impact on splicing (Table S1). Significantly, the c.2278C>T mutational change was observed as a heterozygous change in the patient's mother but not in the father (Figure S3).

**Figure 2:**
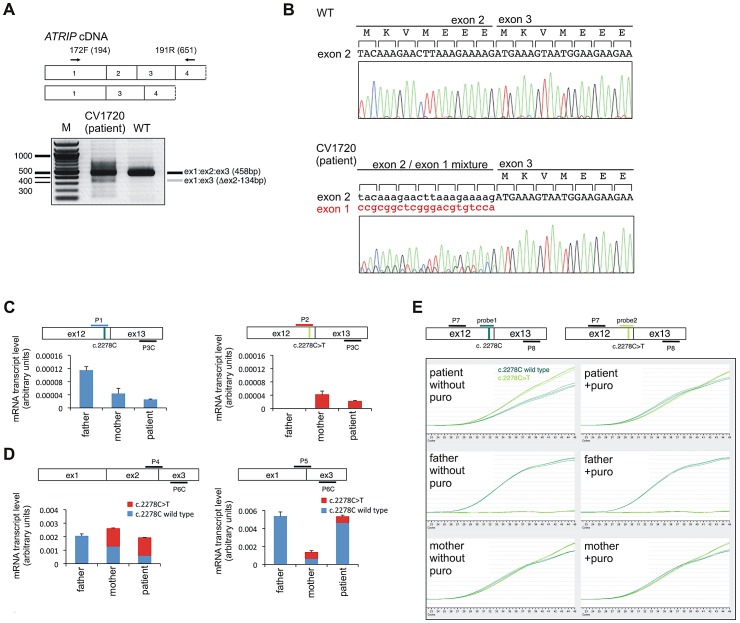
Identification of mutational changes in *ATRIP* in CV1720. A) Upper panel shows primer pairs used to distinguish cDNA products encompassing or lacking exon 2. Lower panel shows RT-PCR products obtained using the primers shown in the upper panel. RT-PCR from patient CV1720 generated a smeared product with a defined band of 458 bp, as observed in WT cells, and a weaker band of 325 bp. The latter band was not detected using cDNA from WT cells (MRC5). A similar single 458 bp band was obtained using the same primers with cDNA derived from a distinct wild type cell line (GM2188; data not shown). (B) Sequencing of the RT-PCR products derived from WT (MRC5) and patient (CV1720) cells. A double sequence pattern at the exon 2–3 boundary is observed using patient CV1720 cDNA. C) Selective quantitative amplification of the WT or 2278C>T *ATRIP* alleles. Primers located in *ATRIP* exon 12 and 13 were designed to selectively amplify the WT (c.2278C) (P1 and P3C) versus the mutated (c.2278C>T) (P2 and P3C) alleles. The WT PCR product is shown in blue and the c.2278C>T PCR product in red. The exon 12 mutated allele is only observed in the patient and mother cDNA whilst the WT allele is observed in the patient, mother and father cDNA although the level is reduced in the patient and mother. D) qRT-PCR analysis of *ATRIP* splicing variants from patient CV1720 and parental cells. qRT-PCR analysis of the level of the normally spliced (encompassing exons 1-2-3) and the aberrantly spliced (Δexon2) *ATRIP* cDNA in the patient and parent cells. PCR primers were designed at the exon2-exon3 or exon1-exon3 boundaries to selectively amplify the splicing variants. Transcripts from *HPRT1* were used as a quantification control. The correctly spliced transcript from the paternal allele of the patient (wild type c.2278C, blue fraction in the cumulative bar labelled, ‘patient’, at the left panel) was estimated to be ∼25% of the normal level. (E) The mis-spliced paternal allele is subject to nonsense mediated mRNA decay (NMD). Cycleave-qPCR confirmed that the *ATRIP* c.2278C>T mutant allele was expressed exclusively in the patient and the mother. The *ATRIP* exon12-13 fragment was amplified with PCR primers P7/P8 as shown in the figure. A set of fluorescent probes were used to distinguish the WT versus c.2278C>T allele (probe1 and probe2, respectively). In the patient, the paternal mRNA transcript level (emerald lines) is low because of NMD (top left). Puromycin treatment eliminated the NMD and the paternal transcript level returned to the normal level. In all panels WT represented MRC5, patient was CV1720 and parents were as shown in Figure 1A.

We also performed RT-PCR sequencing of *ATRIP* cDNA from CV1720 and both parents. These analyses revealed a low level of a smeared PCR product following amplification of the 5′*ATRIP* cDNA region using patient but not control cDNA (data not shown). Following multiple analyses, we found specifically that RT-PCR amplification using primers located in exons 1 and 4, reproducibly yielded a smeared product from CV1720 cDNA with discrete bands at 458 bp (the expected product size) and 325 bp whereas only the 458 bp product was observed using cDNA from WT cells (Figure 2A). Direct sequencing of the gel purified smaller (325 bp) and full-length (458 bp) RT-PCR products showed that the small fragment specifically lacked exon 2. Sequencing analysis of the RT-PCR product of CV1720 cDNA using the same primers revealed the predicted double sequence with the product lacking exon 2 being less than 50% of the product containing exon 2 (Figure 2B). It is notable that there were also some PCR products larger than the full length product although a discrete band was not evident. In sequencing the RT-PCR product, we observed some that harboured intron 2 sequences although these represented a minor product relative to that lacking exon 2. Collectively, these findings strongly suggested that there could be mis-splicing in CV1720 cells with loss of exon 2 being the major product.

To assess this further, qRT-PCR was undertaken using sets of primers that allow selective amplification of the WT and mutant products (c.2278C>T mutant as well as the mis-spliced product). The aim was to determine if the mis-spliced product originated from the paternal allele and if it impacted upon the transcript level. Primer pairs, P1 and P3C, located in exons 12 and 13, respectively, allow selective amplification of the WT (paternal) c.2278C allele whilst primers P2 and P3C selectively amplify the mutated (maternal) c. 2278C>T allele (Figure 2C). As expected, the mutant (c.2278C>T)-allele-specific PCR product (right columns, red bars) was only detected in the patient and mother whereas the WT-specific PCR product (left columns, blue bars) was detected in all samples, demonstrating that the primers distinguished the two alleles (Figure 2C). The results also showed that the c.2278C>T and the WT (c.2278C) alleles were expressed at nearly equal levels (normalised against *HPRT1*) in the mother (compare blue and red bars labelled ‘mother’ in Figure 2C), suggesting that the c.2278C>T *ATRIP* mRNA is not subject to nonsense mediated RNA decay (NMD) (Figure 2C).

To evaluate the expression level of the mis-spliced *ATRIP* mRNA, we designed primers located at the exon 2/exon 3 boundary (primer P4) and within exon 3 (primer 6C) to allow selective amplification of the correctly spliced mRNA (Figure 2D); primers located at the exon 1/exon 3 boundary (primer P5) and within exon 3 (primer 6C) selectively amplify the mis-spliced mRNA. Whilst the correctly spliced product was amplified to similar (although slightly different) extents from father, mother and patient mRNA (compare the column heights, left panel in Figure 2D), the mis-spliced product was more abundant in the patient and father, suggesting that mis-splicing is a consequence of a mutational change linked to the paternal allele (compare the column heights, right panel in Figure 2D). Since we observed nearly equal expression levels of the wild type (c.2278C) and mutant (c.2278C>T) alleles in the mother (Figure 2C, compare the right and left panels), we considered that the PCR products derived from the mother using primers P4/P6C or P5/P6C would be equally derived from the c.2278C and c.2278C>T alleles, which have, therefore, been depicted as equal sized contributions (shown in red or blue in mother columns in Figure 2D). Similarly, the mutant c.2278C>T allele is likely to be expressed at an equal level in the patient as in the mother (shown in red in patient columns in Figure 2D). Based on these assumptions, we estimated that the normally spliced WT mRNA is reduced to around 1/4 of the WT level in the patient and to 3/4 in the father (shown in blue in the left hand panel in Figure 2D). Assuming that the c.2278C>T allele is fully inactivated (see below), the patient therefore has around 25% of ATRIP activity compared to a normal individual.

The findings above suggested that the mis-spliced mRNA, which generates an out of frame cDNA, is subject to NMD. To examine this and substantiate our findings, qRT-PCR was also carried out using fluorescent cycleave probes with or without exposure to puromycin, an antibiotic which prevents NMD (Figure 2E) [29]. Primers (P7 and P8) and fluorescent probes (probe 1 and 2) were designed to allow amplification of a product encompassing exon 12–13 that distinguished the maternal (probe 2) from the paternal (probe 1) allele. We confirmed detection of the c.2278C>T allele exclusively in the patient and mother as well as the WT allele in all samples (Figure 2E). In the mother, the wild type (c.2278C) and mutant (c.2278C>T) signals were detected at equal levels regardless of whether puromycin was added, indicating that the alleles are equally expressed and are not subjected to NMD. In patient CV1720, the WT product was reduced relative to the mutant product in the absence of puromycin but was at similar levels in the presence of puromycin (Figure 2E). These findings are consistent with the notion that the mRNA expressed from the parental allele is aberrantly spliced and partially subject to NMD. Perhaps surprisingly, we did not detect any obvious difference of the WT product following puromycin treatment in the father; however, in this case, we anticipate a 25% decreased product, which is unlikely to be detected without an internal control. However, despite this, there was evidence for abnormal splicing in the paternal cDNA from analysis of the PCR products spanning exons 1–3 (Figure 2B, 2D).

Finally, to gain insight into the basis underlying mis-splicing, we sequenced introns 1 and 2 from the patient, mother, and father and identified a previously unreported mutational change in intron 2, 13 bp from the intron-exon 2 boundary in the patient and paternal gDNA (Table S1). However, given the modest impact on splicing we did not attempt to examine whether this represented the causal mutational change affecting splicing.

### Arg760* ATRIP does not promote ATR–dependent G2/M arrest and reduces ATR–ATRIP interaction

**Figure 3:**
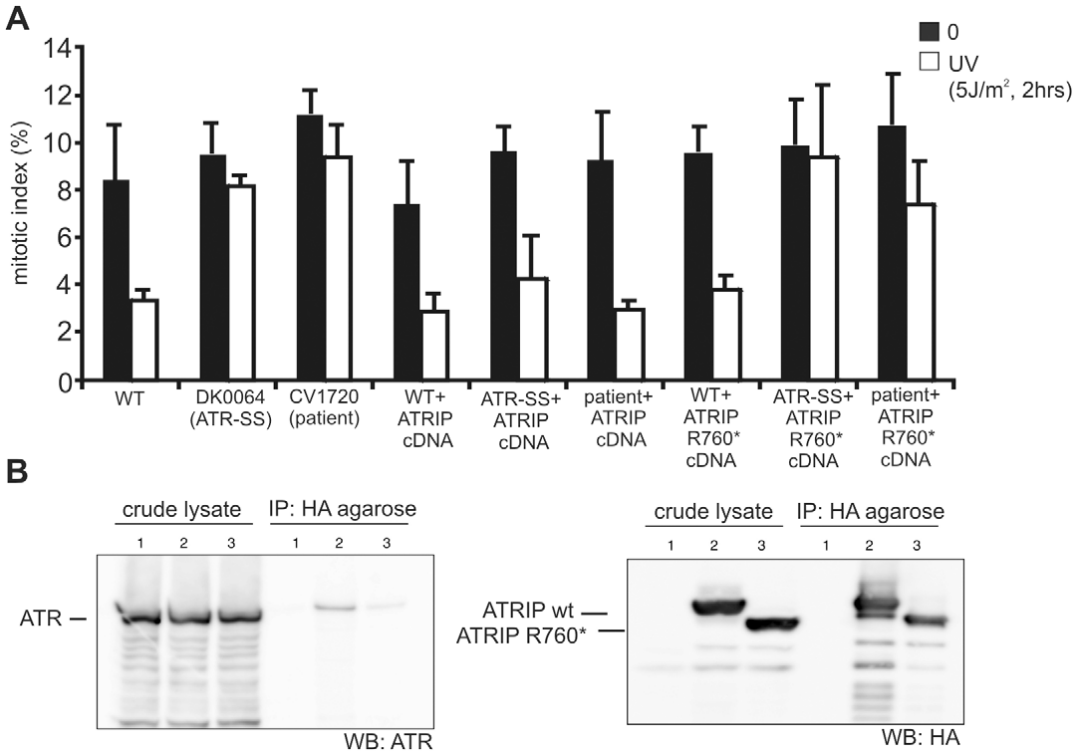
WT ATRIP cDNA but not cDNA encoding p.Arg760* ATRIP complements the G2/M checkpoint defect in CV1720 cells, and p.Arg760*ATRIP impairs ATR–ATRIP protein interaction. A) Analysis of the G2/M checkpoint defect in CV1720 cells following expression of ATRIP cDNA. G2/M checkpoint arrest was examined 2 h post exposure to 5 Jm^−2^ UV. As shown in Figure 1A, WT cells showed proficient checkpoint arrest whilst DK0064 (ATR–SS) and CV1720 (patient) cells are unable to undergo arrest. Expression of WT ATRIP cDNA restored the ability of CV1720 (patient) and DK0064 (ATR–SS) to undergo checkpoint arrest but this was not observed following transfection of cDNA encoding R760* ATRIP. Significantly, expression of ATRIP R760* did not impair checkpoint arrest in WT cells verifying that it does not exert a dominant negative impact. Results represent the mean and SD of three experiments. WT cells were GM2188. ATR–SS represents DK0064 and patient, CV1720. B) R760* ATRIP impairs ATR–ATRIP interaction. Crude lysates were prepared from HEK293T cells and either mock transfected (lane1), transfected with HA-tagged WT *ATRIP* cDNA (lane2), or R760* *ATRIP* cDNA (lane3) (generating p.Arg760* ATRIP protein) together with *ATR* cDNA. The extracts were immunoprecipitated with agarose-conjugated rabbit anti-HA-tag antibody (MBL). Interaction with ATR was examined by immunoblotting with ATR antibodies (left panel). Immunoblotting using the HA-tag (ATRIP; right panel) verified expression of the appropriately sized ATRIP in the samples. 33% of the crude lysate was loaded; IP, immunoprecipitate.

It is likely that *ATRIP* c.2278C>T causes an impacting mutational change since the low levels of ATRIP protein (10–20% WT levels) in CV1720 cells suggest that p.R760* ATRIP is unstable (given that the mRNA level of this allele is normal). To substantiate that p.R760* expression impairs the ATR–dependent response to DNA damage, we examined whether its expression could complement the G2/M checkpoint defect of CV1720 cells. We also examined whether p.R760* might exert a dominant negative impact (since this represented a possible explanation for the low ATRIP protein level in CV1720 cells). The c.2278C>T mutational change was introduced into *ATRIP* cDNA by site directed mutagenesis. cDNA encoding WT *ATRIP* and/or R760* ATRIP was transiently transfected into LBLs and G2/M checkpoint arrest examined at 2 h post exposure to 5 Jm^−2^ UV. Consistent with previous findings, WT but not CV1720 cells showed a G2/M checkpoint arrest (Figure 3A). Whilst transfection with WT *ATRIP* cDNA completely rescued the G2/M checkpoint defect of CV1720 cells, no correction was observed in CV1720 cells following expression of c.2278C>T *ATRIP* cDNA (encoding R760* ATRIP). Surprisingly, expression of WT *ATRIP* cDNA also corrected the G2/M checkpoint defect in DK0064 (ATR–SS) cells, which we propose could result from elevated ATRIP expression causing stabilisation of residual ATR protein, since ATR–SS cells have low ATR and ATRIP expression. Significantly, c.2278C>T *ATRIP* cDNA was unable to rescue ATR–SS cells. Finally expression of c.2278C>T *ATRIP* cDNA in WT cells did not affect G2/M checkpoint arrest demonstrating that p.R760* ATRIP does not exert a dominant negative impact. Collectively, we conclude that p.R760* ATRIP impacts upon ATRIP function.

Next we examined how loss of the ATRIP C-terminus might impact upon ATRIP function. Two studies have previously observed that the C-terminal region of ATRIP is required for interaction with ATR [21], [30]. Falck *et al* [30] reported that ATR–ATRIP interaction required the C-terminal 32 amino-acids of ATRIP (769–791) whilst Ball *et al* [21] found that interaction was abolished in a protein that lacked exon 11, which encompasses amino-acids 658–684. Arg760 lies close to these regions. To examine whether p.R760* ATRIP can interact with ATR, HA-tagged *WT* or c.2278C>T (ATRIP R760*) cDNA was co-expressed with untagged WT *ATR* cDNA in HEK293 cells. Following IP with HA-agarose, the level of co-immunoprecipitated ATR was assessed by Western Blotting. Although there was a low level of non-specific ATR binding to the HA beads, the level of ATR present after HA-R760* ATRIP expression (derived from c.2278C>T *ATRIP* cDNA) was substantially lower than after HA-WT ATRIP expression (Figure 3B left panel). Both WT and R760* ATRIP were efficiently expressed, however (Figure 3B right panel). Thus, we conclude that R760* impairs the binding of ATRIP to ATR.

### Identification of further patients with mutations in *ATR*

**Figure 4:**
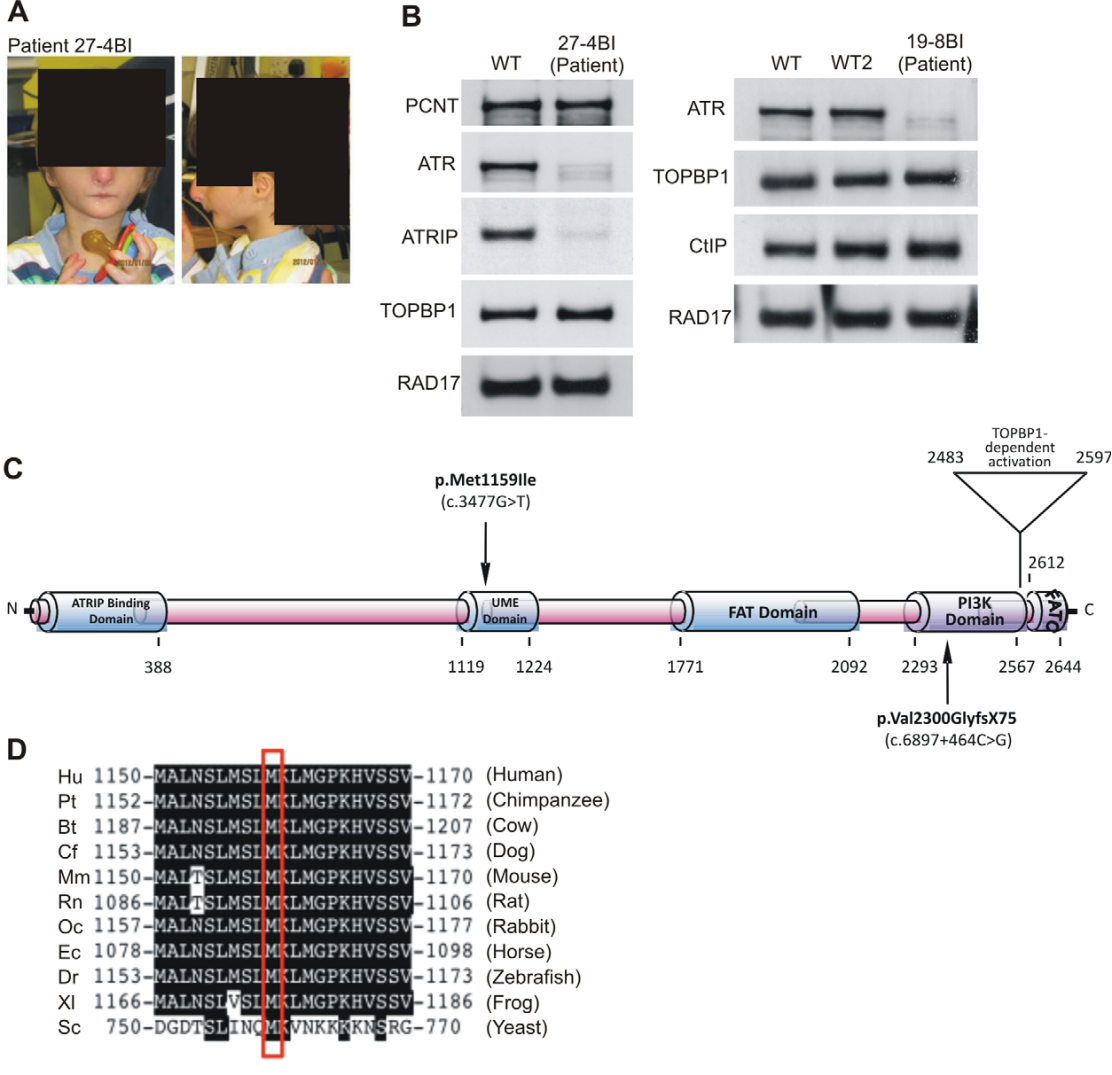
Patients 27-4BI and 19-8BI have reduced ATR and ATRIP expression and mutations in ATR. A) Photographs of patient included with informed consent of parent. B) Cell extracts (50 µg) from LBLs derived from WT (IM257), patient 27-4BI or patient 19-8BI were immunoblotted using the indicated antibodies. Reduced expression of ATR was observed in both patients. 27-4BI also had reduced ATRIP expression. C) Structure of ATR showing the site of the mutations identified and the UME domain. D) The UME domain is conserved between species and the methionine residue within this domain is conserved in yeast.

In the course of our functional characterisation of cell lines from SS patients, we examined LBLs derived from two SS patients, 27-4BI and 19-8BI (see Figure 4A, Figure S4, and Table 1 for clinical details). Western Blotting revealed that both cell lines displayed substantially reduced ATR protein whilst showing normal expression of other DNA damage response components, including CtIP, TOPBP1 and RAD17 (Figure 4B). 27-4BI also had reduced ATRIP levels. Additionally, the 27-4BI cell line expressed normal levels of PCNT, excluding MOPD type II as a potential genetic diagnosis, since most of these patients exhibit severely reduced PCNT expression. These findings raised the possibility that the patients could harbour mutations affecting ATR or ATRIP expression. Sequencing of *ATR* cDNA revealed the same c.3477G>T mutational change in both patients (Figure S5A). This change causes an amino acid substitution, p.Met1159Ile, which lies within a conserved UME (NUC010) domain of ATR. UME domains, and particularly the methionine residue within the domain, are highly conserved in ATR species, including yeast although their function is unknown (Figure 4C and 4D).

The second *ATR* mutation identified was c.6897+464C>G;p.Val2300Gly fs75*, which, surprisingly, was also present in both patients. RT-PCR sequencing showed that a 142 bp sequence, which originated from a repeat region present in intron 40, was inserted at the boundary between exon 40 and 41 in both patients (Figure S5C). Genomic sequencing revealed the presence of a single C>G mutation in intron 40, which generates a preferred splice signal causing insertion of the intron sequence to the start of exon 41 (Figure S5D for further details). This insertion causes a frameshift and the generation of a stop codon at c.6978 in exon 41. Sequencing of *ATRIP* cDNA in patient 27-4BI failed to reveal any mutational changes. Thus, our findings provide strong evidence that mutational changes in ATR underlie the reduced ATR/ATRIP expression observed in both patients.

**Figure 5:**
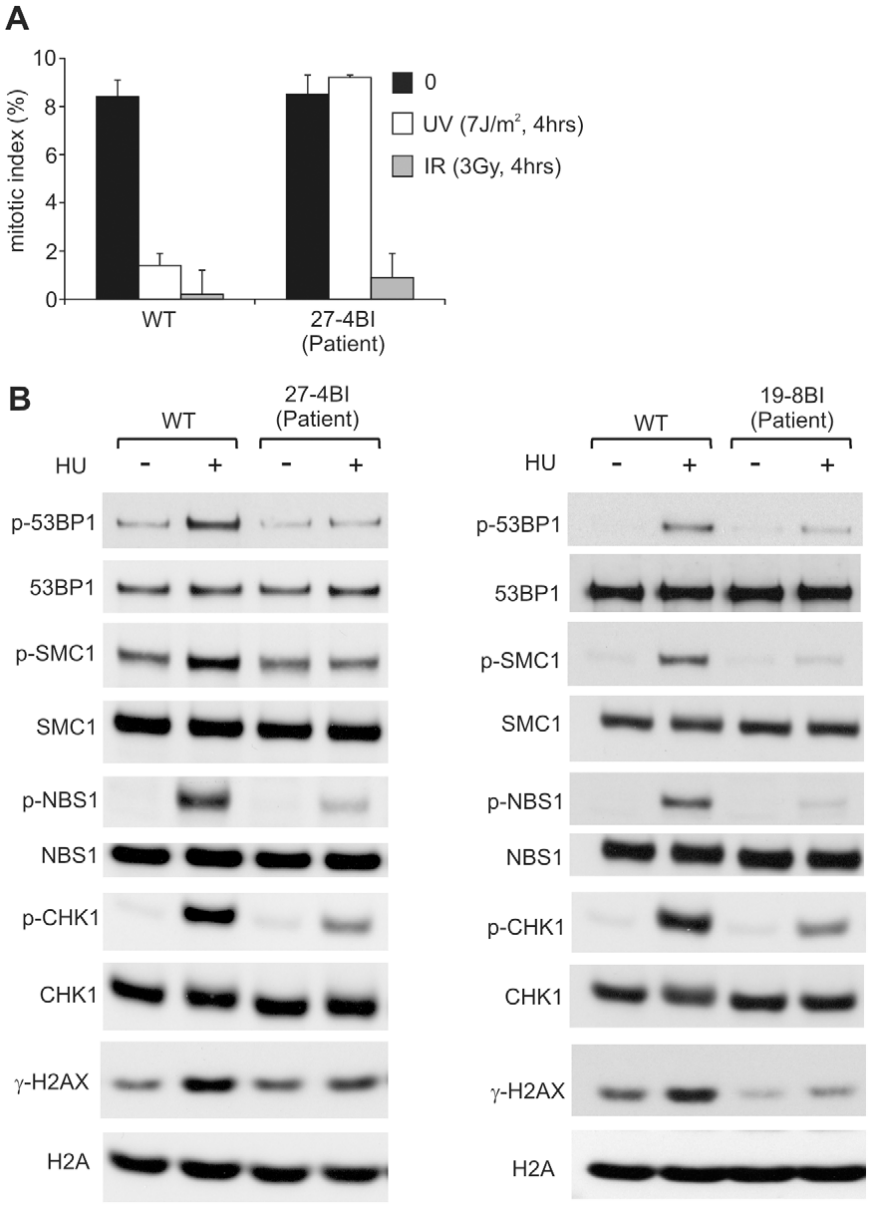
LBLs from patient 27-4BI and 19-8BI showed impaired ATR–dependent damage responses. A) 27-4BI cells were examined for their ability to activate G2/M checkpoint arrest at 4 h following exposure to 7 Jm^−2^ UV. In contrast to WT cells (GM2188), no significant arrest was observed in 27-4BI cells. The checkpoint response to ionizing radiation, which is ATM rather than ATR dependent, was normal. B) LBLs derived from patients 27-4BI and 19-8BI were examined for their ability to phosphorylate the indicated ATR substrates at 1 h following exposure to 0.5 mM HU. WT represents IM257. 27-4BI and control LBLs have a similar cell cycle profile demonstrating that the lack of ATR substrate phosphorylation cannot be attributed to the lack of S phase cells (Figure S2).

To verify that these mutational changes impact upon ATR function, we examined whether 27-4BI cells could activate UV-induced ATR–dependent G2/M checkpoint arrest. Significantly, we observed an inability to activate UV-induced G2/M checkpoint arrest in 27-4BI cells similar to that observed in DK0064 (ATR–SS) cells (Figure 5A) [7]. Checkpoint arrest after exposure to ionising radiation was activated normally. Additionally, we examined the phosphorylation of a range of ATR substrates following exposure to 0.5 mM HU and observed impaired phosphorylation in both 27-4BI and 19-8BI cells (Figure 5B). Collectively, these functional data substantiate a deficiency in the ATR–dependent DNA damage response in LBLs from these two cases. Thus, we conclude that both patients represent further ATR–SS patients.

## Discussion

Although the first causal defect for SS was identified as ATR in 2003, further patients with mutations in *ATR* have not been reported [7]. SS patients are characterised by microcephaly and growth delay, features also observed in other microcephalic, primordial dwarfism syndromes including MOPD type II and MGS. Given that all ATR–SS patients to date share consanguinity, there are limitations in defining the spectrum of clinical features conferred by ATR deficiency to support a clinical distinction between ATR–SS and related disorders such as MOPD type II and MGS as well as other sub-classes of SS [1]–[3], [31].

Here, we describe the novel identification of a patient mutated in ATRIP, the binding partner of ATR. Thus, we identify *ATRIP* as a new causal gene for SS. The mutational change in one *ATRIP* allele lies within a region previously suggested to be required for interaction with ATR, which is consolidated by our work [21], [30]. We demonstrate that the second allele is abnormally spliced causing a reduction in *ATRIP* mRNA from that allele. qRT-PCR analysis suggested that there could be 25% residual WT *ATRIP* expressed in the patient cells. Consistent with this, we routinely observed ∼10–20% of WT ATRIP protein in CV1720 cells by Western Blotting, although the level was variable between preparations. Although not examined in detail, there appeared to be a correlation between proliferation status and ATRIP levels, with the levels decreasing as proliferation slowed. Thus, differences in the proliferative state of cells at the time of analysis may underlie the apparent difference between Western Blotting and qRT-PCR analysis. Notwithstanding some limitations in quantification, the patient clinical features were marked despite ∼10–20% residual ATRIP expression. Similarly, in patient DK0064, residual ATR protein can be readily detected [7]. Thus, we conclude that reduced but detectable levels of ATR/ATRIP protein can confer a clinical phenotype.

Additionally, we identify two further SS patients with ATR mutations in two unrelated families native to the UK. Interestingly, despite being unrelated, 27-4BI and 19-8BI carry the same compound heterozygous mutations, possibly representing founder mutations in the UK population.

All four ATR/ATRIP patients displayed severe microcephaly and growth delay (Table 1). All patients also displayed micrognathia, receding forehead, dental crowding and microtia with small or absent lobes (Figure 4A). Interestingly, an MRI scan of the ATRIP–SS patient revealed an abnormally small pituitary with absent fossa, which could contribute to the delayed growth observed (Figure S1). In distinction to the original ATR–SS patient (DK0064), patients 27-4BI and 19-8BI showed more marked skeletal abnormalities including digital features and aberrant patellae suggesting that ATR deficiency can have a detrimental impact on bone development (Table 1, Figure S4) [32]. Interestingly, aberrant patellae is a clinical feature commonly exhibited by MGS patients suggestive of a biological overlap between the ATR checkpoint pathway and the replication machinery during skeletal development and maintenance. In keeping with this, characterisation of a mouse model harbouring the same mutational change identified in the original ATR–SS patient (DK0064) revealed marked bone abnormalities including kyphosis and osteoporosis [32]. Our findings suggest that ATR–ATRIP SS shows more overlap with MGS than previously recognised (Table 2). However, whereas ATR–ATRIP SS patients tend to have very marked microcephaly, growth delay, dental crowding, small ears and less severe skeletal abnormalities, the spectrum for MGS tends to be less marked microcephaly and growth delay but a striking impact on skeletal development. Nonetheless, there does not appear to be an absolute clinical divide between these two disorders. Significantly, these overlapping clinical features could reflect the fact that both ATR/ATRIP and the origin licensing complex play an essential role in promoting efficient replication and recovery from fork stalling, which may be vital during developmental stages involving rapid replication [13].

**Table 2:** MGS and Seckel syndrome patient phenotypes.

	ORC1 - MGS	Pre-RC MGS	ATR/ATRIP SS
Number of patients	10	25	4
OFC (cm)*	−5.4 to −11 SD	+1.7 to −5.0 SD	−10 to −12 SD
Height (cm)*	−4.5 to −9.6 SD	−0.4 to −6.4 SD	−5 to −8 SD
Weight (kg)*	0.8 to −11 SD	−0.3 to −9.9 SD	−3.3 to −8 SD
Intellectual disability	Ranges from none to mild/moderate	None	Developmental delay (2/4)
Facial Features	Small and abnormal ears (9/10), micrognathia (5/10), down slanted palpebral fissures (1/10)	Small and abnormal ears(25/25), micrognathia (20/25), down slanted palpebral fissures (8/25)	Small and/or abnormal ears (4/4), micrognathia (4/4), receding forehead (4/4), prominent nose (4/4), short palpebral fissures (2/4)
Skeletal abnormalities	Delayed bone age (3/10), Slender long bones (2/10) , absent patellae (6/10), genu recurvatum (4/10)	Delayed bone age (11/25), slender long bones, absent patellae (24/25)	Delayed bone age (1/4), 5^th^ finger clinodactyly (2/4), symmetric dwarfism (3/4), small/abnormal patellae (2/4), kyphosis (1/4), hip abnormality (2/4), narrow pelvis (iliac blades) (1/4)
MRI	Normal in 2 patients examined	NA	Generalised cerebral atrophy, delayed myelination, abnormal gyration (2 patients examined)
Other	High pitched voice (1/10), full lips (7/10), cryptochordism (2/4 examined), mammary hypoplasia (2/2 examined), feeding and respiratory problems during infancy (8/10)	Full lips (14/25), cryptochordism (7/14 examined), mammary hypoplasia (8/8 examined), feeding (20/25) and respiratory (9/25) problems during infancy	Dental crowding (4/4), feeding and respiratory problems during infancy (1/4)

* standard deviations from the age-related normal population mean, NA = not assessed.

MGS data from [13], [14] [33], [34].

In summary, we provide the first report of a SS patient with mutations in *ATRIP*, defining a further novel genetic defect for this disorder, and describe two additional patients native to the UK, with mutations in *ATR*. The description of multiple ATR–ATRIP patients allows us to define a spectrum of clinical features conferred by *ATR*–*ATRIP* mutations. The clinical characteristics include severe microcephaly and growth delay, small or absent ear lobes, micrognathia and dental crowding. In addition, the novel ATR–mutated cases described here expand the clinical impact of impaired ATR–function to include more marked skeletal involvement.

## Methods

### Ethics Statement

Ethical approval for the research was granted by the School of Life Sciences Research Governance Committee, University of Sussex. Informed consent was obtained and clinical investigations were conducted according to the principles expressed in the Declaration of Helsinki. Patient material was gathered under conditions of the Human Tissue Authority (HTA licence number 12119).

### Patients and cell lines

CV1720 is a SS patient of Gujarati-Indian origin. Patients 27-4BI and 19-8BI are English. The clinical features are described in Table 1. Lymphoblastoid cell lines (LBLs) were derived from blood following EBV transformation. WT LBLs were GM2188 or LB197 as indicated. All LBLs were grown in RPMI medium supplemented with 10% foetal calf serum, penicillin, and streptomycin. Transfection with *ATRIP* cDNA was with Genejuice Transfection Reagent (Novagen, Merck Millipore, UK) following the manufacturers protocol.

#### qRT–PCR

Transcript levels of the *ATRIP–*c.2278C (normal) and *ATRIP–*c.2278C>T (p.Arg760*) alleles in LBLs from patient CV1720, and the parents were determined by the cycleave quantitative real time PCR (Cycleave-qPCR, TaKaRa Co. Ltd, Kyoto Japan) as well as standard site specific q-PCR (carried out in triplicate). Transcripts from the *HPRT1* allele were used as a quantification control. In the Cycleave qPCR, RNaseH sensitive fluorescent probes that specifically recognize the c.2278C and c.2278C>T alleles were used for the assay. qPCR results were analyzed by the ΔΔCT method. qPCR primers and probes used for the assay are listed below. (172F, 5′-CTTCACTGCCGACGACCTGG-3′; 191R, 5′-TTTGCTCGTTCACTGGTCTG-3′; P1, 5′-GGGGTCAGCATGCTCATCC-3′; P2, 5′-GGGGGTCAGCATGCTCATCT-3′; P3C, 5′-ACCTCGGGGTCTTCCACATC-3′; P4, 5′- -3′; P5, 5′- -3′; P6C, 5′- -3′; P7, 5′-GCCTATCGCAGAAGGACAAG-3′; P8, 5′-GGGTCTTCCACATCGGTTTC-3′; probe1 for c.2278C, 5′Eclipse-CCCTC(rG)GAT-3′FAM; probe2 for c.2278C, 5′Eclipse- GCCCTC(rA)GA-3′ROX)

#### Co-immunoprecipitation

To investigate the interaction of the ATRIP proteins with ATR, HEK293T cells were transfected with the HA-tagged *ATRIP* cDNA expressing plasmids (wild type and 2278C>T *ATRIP*) together with *ATR* cDNA, followed by 24 h incubation. Whole cell lysates were prepared using CelLytic Nuclear Extraction Kit (Sigma, St. Louis). Co-immunoprecipitation was performed using rabbit anti-HA antibody-conjugated agarose beads (MBL, Nagoya, Japan). Western blotting was carried out using ATR or anti-HA (detecting HA-tagged ATRIP) antibodies. Anti-ATR was N19 (Santa Cruz, Santa Cruz) at 1∶200 dilution. Anti-HA-tag antibody, 132-3 (MBL, Nagoya, Japan), was used at 1∶1000 dilution.

### Immunofluorescence for analysis of γH2AX and 53BP1 staining

Cells were cytospun onto slides, fixed with 3% formaldehyde for 10 min and permeabilized in 0.5% Triton-X100. After antibody treatment and staining with 4,6-diamidino-2-phenylindole (DAPI), coverslips were mounted in Vectashield mounting medium (Vector Laboratories, Burlingame). Samples were incubated with primary antibodies for γ-H2AX (Millipore, Billerica) or 53BP1 (Bethyl, Montgomery). Secondary antibodies were from Sigma (St. Louis).

### Western blotting

Cells were lysed for one hour in IPLB (50 mM Tris-HCl, 150 mM NaCl, 2 mM EDTA, 2 mM EGTA, 25 mM NaF, 25 mM β-glycerolphosphate, 0.1 mM NaOrthovanadate, 0.2% Triton X-100, 0.3% NP-40, plus protease inhibitor cocktail (Roche, Basel) at 4°C, centrifuged at 13,000 rpm for 10 minutes. The soluble fraction was subjected to SDS-PAGE and transferred to a nitrocellulose membrane for protein detection.

Antibodies raised against ATR, CHK1 (FL476) and MCM2 (N19) were from Santa Cruz (Santa Cruz). Anti-FANCD2, ATRIP and phospho-Chk1 (Ser317) antibodies were from Novus (Littleton), Bethyl (Montgomery), and Cell Signaling (Beverly, Woburn), respectively.

### G2/M checkpoint arrest

Cells were exposed to 5 or 7 Jm^−2^ UV, or 3Gy ionising radiation and incubated for 2 or 4 hours (as indicated) in complete medium containing 0.2 ug/ml Colcemid (Invitrogen, Carlsbad), followed by processing for immunofluorescence as detailed above. Mitotic cells were detected by α-Histone H3-pSer10 antibodies (Millipore, Billerica) and cells were counterstained with DAPI.

## Supporting Information

Figure S1 Photograph of limbs and MRI scan of patient CV1720. Left hand photograph showing hands and feet. Right hand photograph shows an MRI scan where a small pituitary is evident.
(TIF)

Figure S2 Cell cycle analysis of WT, DK0064 (ATR–SS), 27-4BI, and CV1720 patient LBLs. A) Asynchronous growing cultures of WT, DK0064 (ATR–SS), 27-4BI and CV1720 patient cells were pelleted, fixed in 70% ice-cold ethanol and stained with propidium iodide prior to FACs analysis. Populations were gated and the proportion of cells in G1, S and G2/M phases of the cell cycle measured. B) WT, DK0064 (ATR–SS), 27-4BI and CV1720 patient cells were treated with nocodazole for 16 h and then fixed and analysed as in a). C) Asynchronous growing cultures of WT, DK0064 (ATR–SS), 27-4BI and CV1720 patient cells were pulse-labelled with 50 µM BrdU for 1 h. Cells were then fixed in 70% ice-cold ethanol and processed for BrdU FACs analysis as described in Bicknell et al, 2011 [13]. The proportion of cells in S phase were gated and measured. Each graph represents the mean of three independent experiments. The error bars represent the standard deviation.
(TIF)

Figure S3 Identification of a truncating mutation in *ATRIP* in patient, CV1720. Genomic DNA sequencing of *ATRIP* exons showed that patient CV1720 and the unaffected mother, CV1780, are heterozygous for a c.2278C>T mutational change in exon12 of *ATRIP*. The father has a WT sequence at this site. 2278C>T generates a primary stop codon predicting a truncated protein at position arginine 760 (p.R760*). WT sequence shown in blue, the mutation is shown in Red.
(TIF)

Figure S4 Photographs of patients 27-4BI and 19-8BI. A) Shows abnormal digits of patient 27-4BI. B) Copper beaten appearance of skull of patient 19-8BI. C) Frontal and Lateral view of left knee of patient 19-8BI showing an absence of ossification of the patella.
(TIF)

Figure S5 Mutational changes observed in *ATR* in patients 27-4BI and 19-8BI. A) c.3477G>T mutational change in patients 27-4BI and 19-8BI. RT-PCR sequencing revealed a heterozygous 3477G>T mutational change in both patients causing an amino acid substitution, p.Met1159Ile, which lies within a conserved UME (NUC010) domain of ATR. B) A double sequence was observed at the boundary between exons 40 and 41 in both patients. Sequencing showed that the double sequence was caused by insertion of a 142 bp region from intron 40. C) A C to G mutational change was observed in intron 41 of both patients converting the sequence CAGCT to CAGGT, a splice site. The insertion causes a frameshift and a stop codon at p.Val2300Glyfs*75. D) Diagram showing the likely origin of the insertion observed at the exon 40/41 boundary. Sequencing of intron 40 revealed a C>G mutation as indicated creating a cryptic splice site causing splicing of exon 40 to the indicated intronic sequence (which represents an Alu repeat sequence). Thus one ATR allele of the patients harbours a 142 nucleotide insertion between exons 40 and 41. Exons 40 and 41 are highlighted in green and the inserted intronic sequence is shown in red. The intronic C>G change is highlighted in red. The insertion causes a frameshift and a stop codon at c.6978 in exon 41.
(TIF)

Table S1 The table shows the position of single nucleotide polymorphisms identified in intron 1 and 2 in the patient and parental genomic DNA. ^*^ The contig position is defined as the position of the single nucleotide variant (SNV) on the contig (NT_022517.17) when counting from the first base (base position = 1). ^**^rs# is the NCBI's reference SNP ID. ^***^ minor allele (indicated as a base) and its frequency (MAF) (second most frequent allele) in a default global population reported in dbSNP database (1000 Genome phase 1, May 2011). N.A. not available.
(DOCX)

## References

[1] Majewski F, Goecke T (1982) Studies of microcephalic primordial dwarfism I: approach to a delineation of the Seckel syndrome. Am J Med Genet 12: 7–21. 7046443 10.1002/ajmg.1320120103

[2] Hall JG, Flora C, Scott CI Jr, Pauli RM, Tanaka KI (2004) Majewski osteodysplastic primordial dwarfism type II (MOPD II): natural history and clinical findings. Am J Med Genet A 130: 55–72. 10.1002/ajmg.a.30203 15368497

[3] Gorlin RJ (1992) Microtia, absent patellae, short stature, micrognathia syndrome. J Med Genet 29: 516–517. PMC1016043 1640440

[4] Thornton GK, Woods CG (2009) Primary microcephaly: do all roads lead to Rome? Trends Genet 25: 501–510. 19850369 10.1016/j.tig.2009.09.011 PMC2816178

[5] Goodship J, Gill H, Carter J, Jackson A, Splitt M, et al (2000) Autozygosity mapping of a seckel syndrome locus to chromosome 3q22. 1-q24. Am J Hum Genet 67: 498–503. 10889046 10.1086/303023 PMC1287195

[6] Borglum AD, Balslev T, Haagerup A, Birkebaek N, Binderup H, et al (2001) A new locus for Seckel syndrome on chromosome 18p11.31-q11.2. Eur J Hum Genet 9: 753–757. 11781686 10.1038/sj.ejhg.5200701

[7] O'Driscoll M, Ruiz-Perez VL, Woods CG, Jeggo PA, Goodship JA (2003) A splicing mutation affecting expression of ataxia-telangiectasia and Rad3-related protein (ATR) results in Seckel syndrome. Nature Genetics 33: 497–501. 12640452 10.1038/ng1129

[8] Qvist P, Huertas P, Jimeno S, Nyegaard M, Hassan MJ, et al (2011) CtIP Mutations Cause Seckel and Jawad Syndromes. PLoS Genet 7: e1002310 doi:10.1371/journal.pgen.1002310. 21998596 10.1371/journal.pgen.1002310 PMC3188555

[9] Al-Dosari MS, Shaheen R, Colak D, Alkuraya FS (2010) Novel CENPJ mutation causes Seckel syndrome. J Med Genet 47: 411–414. 20522431 10.1136/jmg.2009.076646

[10] Kalay E, Yigit G, Aslan Y, Brown KE, Pohl E, et al (2011) CEP152 is a genome maintenance protein disrupted in Seckel syndrome. Nat Genet 43: 23–26. 21131973 10.1038/ng.725 PMC3430850

[11] Griffith E, Walker S, Martin CA, Vagnarelli P, Stiff T, et al (2008) Mutations in pericentrin cause Seckel syndrome with defective ATR–dependent DNA damage signaling. Nat Genet 40: 232–236. 18157127 10.1038/ng.2007.80 PMC2397541

[12] Rauch A, Thiel CT, Schindler D, Wick U, Crow YJ, et al (2008) Mutations in the pericentrin (PCNT) gene cause primordial dwarfism. Science 319: 816–819. 18174396 10.1126/science.1151174

[13] Bicknell LS, Walker S, Klingseisen A, Stiff T, Leitch A, et al (2011) Mutations in ORC1, encoding the largest subunit of the origin recognition complex, cause microcephalic primordial dwarfism resembling Meier-Gorlin syndrome. Nat Genet 43: 350–355. 21358633 10.1038/ng.776

[14] Bicknell LS, Bongers EM, Leitch A, Brown S, Schoots J, et al (2011) Mutations in the pre-replication complex cause Meier-Gorlin syndrome. Nat Genet 43: 356–359. 21358632 10.1038/ng.775 PMC3068194

[15] Willems M, Genevieve D, Borck G, Baumann C, Baujat G, et al (2010) Molecular analysis of pericentrin gene (PCNT) in a series of 24 Seckel/microcephalic osteodysplastic primordial dwarfism type II (MOPD II) families. Journal of medical genetics 47: 797–802. 19643772 10.1136/jmg.2009.067298

[16] Nam EA, Cortez D (2011) ATR signalling: more than meeting at the fork. The Biochemical journal 436: 527–536. 21615334 10.1042/BJ20102162 PMC3678388

[17] Zou L, Elledge SJ (2003) Sensing DNA damage through ATRIP recognition of RPA-ssDNA complexes. Science 300: 1542–1548. 12791985 10.1126/science.1083430

[18] Ciccia A, Elledge SJ (2010) The DNA damage response: making it safe to play with knives. Mol Cell 40: 179–204. 20965415 10.1016/j.molcel.2010.09.019 PMC2988877

[19] Cortez D, Guntuku S, Qin J, Elledge SJ (2001) ATR and ATRIP: partners in checkpoint signaling. Science 294: 1713–1716. 11721054 10.1126/science.1065521

[20] Namiki Y, Zou L (2006) ATRIP associates with replication protein A-coated ssDNA through multiple interactions. Proceedings of the National Academy of Sciences of the United States of America 103: 580–585. 16407120 10.1073/pnas.0510223103 PMC1334680

[21] Ball HL, Myers JS, Cortez D (2005) ATRIP binding to replication protein A-single-stranded DNA promotes ATR–ATRIP localization but is dispensable for Chk1 phosphorylation. Molecular biology of the cell 16: 2372–2381. 15743907 10.1091/mbc.E04-11-1006 PMC1087242

[22] Paciotti V, Clerici M, Lucchini G, Longhese MP (2000) The checkpoint protein Ddc2, functionally related to S. pombe Rad26, interacts with Mec1 and is regulated by Mec1-dependent phosphorylation in budding yeast. Genes Dev 14: 2046–2059. 10950868 PMC316858

[23] Lakin ND, Jackson SP (1999) Regulation of p53 in response to DNA damage. Oncogene 18: 7644–7655. 10618704 10.1038/sj.onc.1203015

[24] Ward IM, Chen J (2001) Histone H2AX is phosphorylated in an ATR–dependent manner in response to replicational stress. J Biol Chem 276: 47759–47762. 11673449 10.1074/jbc.C100569200

[25] Alderton GK, Joenje H, Varon R, Borglum AD, Jeggo PA, et al (2004) Seckel syndrome exhibits cellular features demonstrating defects in the ATR signalling pathway. Human Molecular Genetics 13: 3127–3138. 15496423 10.1093/hmg/ddh335

[26] Toledo LI, Murga M, Zur R, Soria R, Rodriguez A, et al (2011) A cell-based screen identifies ATR inhibitors with synthetic lethal properties for cancer-associated mutations. Nature structural & molecular biology 18: 721–727. 10.1038/nsmb.2076 PMC4869831 21552262

[27] Chanoux RA, Yin B, Urtishak KA, Asare A, Bassing CH, et al (2009) ATR and H2AX cooperate in maintaining genome stability under replication stress. The Journal of biological chemistry 284: 5994–6003. 19049966 10.1074/jbc.M806739200 PMC2645842

[28] Andreassen PR, D'Andrea AD, Taniguchi T (2004) ATR couples FANCD2 monoubiquitination to the DNA damage response. Genes and Development 18: 1958–1963. 15314022 10.1101/gad.1196104 PMC514175

[29] Noensie EN, Dietz HC (2001) A strategy for disease gene identification through nonsense-mediated mRNA decay inhibition. Nature biotechnology 19: 434–439. 10.1038/88099 11329012

[30] Falck J, Coates J, Jackson SP (2005) Conserved modes of recruitment of ATM, ATR and DNA-PKcs to sites of DNA damage. Nature 434: 605–611. 15758953 10.1038/nature03442

[31] Klingseisen A, Jackson AP (2011) Mechanisms and pathways of growth failure in primordial dwarfism. Genes & development 25: 2011–2024. 21979914 10.1101/gad.169037 PMC3197200

[32] Murga M, Bunting S, Montana MF, Soria R, Mulero F, et al (2009) A mouse model of ATR–Seckel shows embryonic replicative stress and accelerated aging. Nature genetics 41: 891–898. 19620979 10.1038/ng.420 PMC2902278

[33] Guernsey DL, Matsuoka M, Jiang H, Evans S, Macgillivray C, et al (2011) Mutations in origin recognition complex gene ORC4 cause Meier-Gorlin syndrome. Nat Genet 43: 360–364. 21358631 10.1038/ng.777

[34] de Munnik SA, Bicknell LS, Aftimos S, Al-Aama JY, van Bever Y, et al (2012) Meier-Gorlin syndrome genotype-phenotype studies: 35 individuals with pre-replication complex gene mutations and 10 without molecular diagnosis. European journal of human genetics : EJHG 20: 598–606. 22333897 10.1038/ejhg.2011.269 PMC3355263

