# Supplementary figures and images for: Identification of the First ATRIP–Deficient Patient and Novel Mutations in ATR Define a Clinical Spectrum for ATR–ATRIP Seckel Syndrome
Source: PLoS Genet. 2012 Nov 8;8(11):e1002945. doi: 10.1371/journal.pgen.1002945 (PMC3493446; doi:10.1371/journal.pgen.1002945)

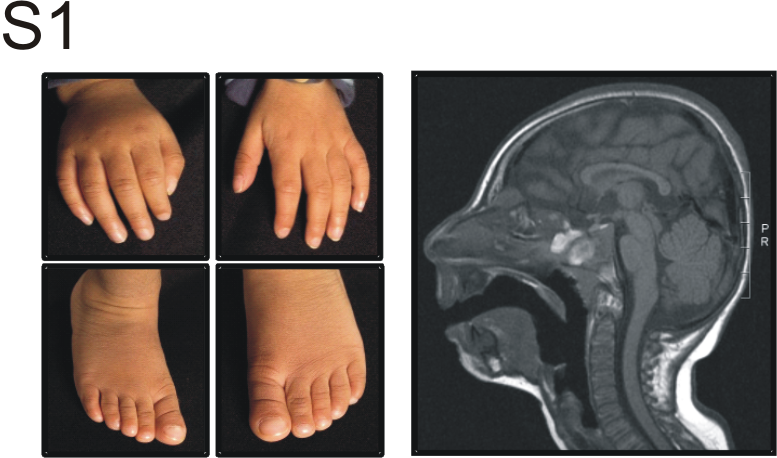

Supplement: Figure S1 — Photograph of limbs and MRI scan of patient CV1720. Left hand photograph showing hands and feet. Right hand photograph shows an MRI scan where a small pituitary is evident. (TIF) [file pgen.1002945.s001.tif]

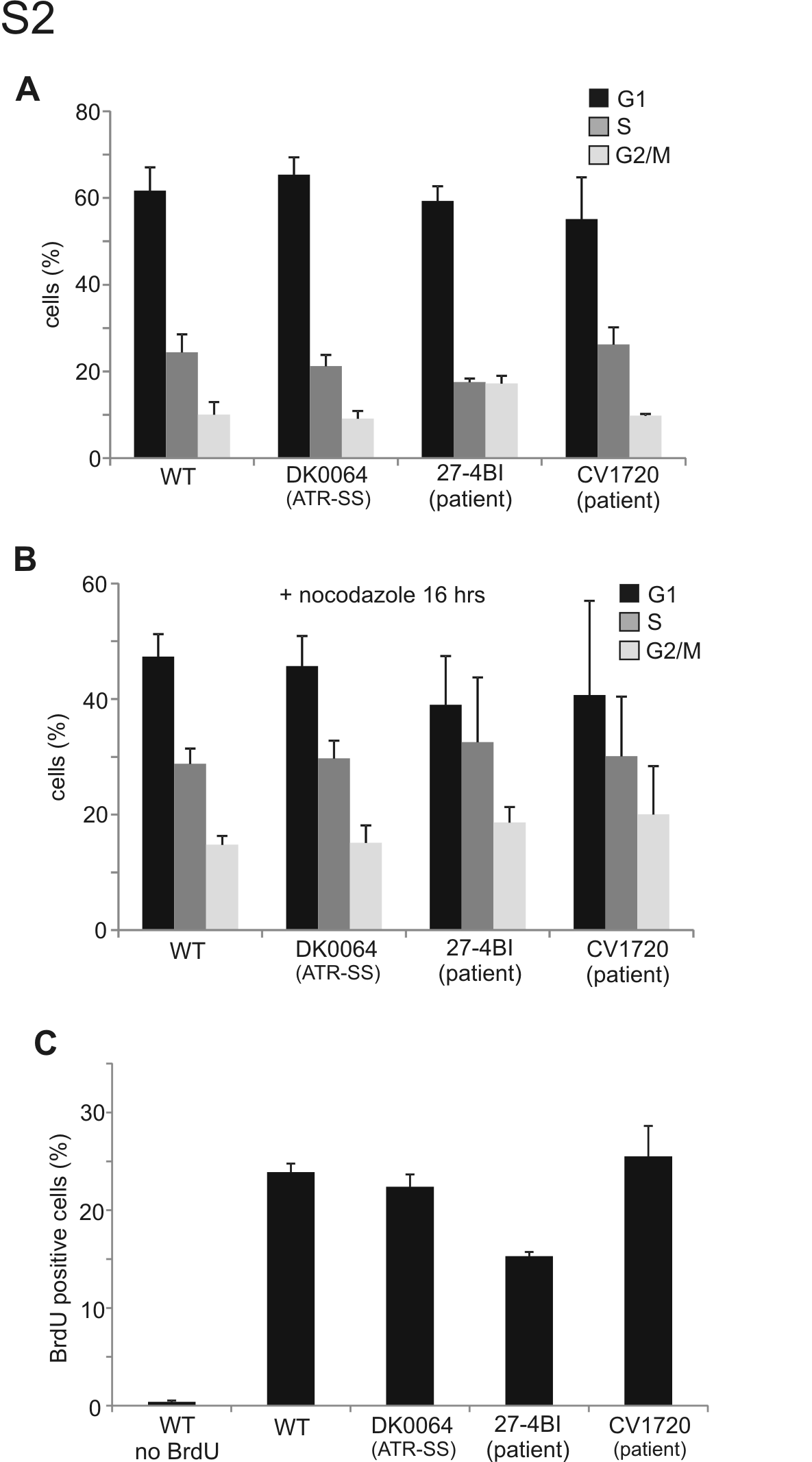

Supplement: Figure S2 — Cell cycle analysis of WT, DK0064 (ATR–SS), 27-4BI, and CV1720 patient LBLs. A) Asynchronous growing cultures of WT, DK0064 (ATR–SS), 27-4BI and CV1720 patient cells were pelleted, fixed in 70% ice-cold ethanol and stained with propidium iodide prior to FACs analysis. Populations were gated and the proportion of cells in G1, S and G2/M phases of the cell cycle measured. B) WT, DK0064 (ATR–SS), 27-4BI and CV1720 patient cells were treated with nocodazole for 16 h and then fixed and analysed as in a). C) Asynchronous growing cultures of WT, DK0064 (ATR–SS), 27-4BI and CV1720 patient cells were pulse-labelled with 50 µM BrdU for 1 h. Cells were then fixed in 70% ice-cold ethanol and processed for BrdU FACs analysis as described in Bicknell et al, 2011 [13]. The proportion of cells in S phase were gated and measured. Each graph represents the mean of three independent experiments. The error bars represent the standard deviation. (TIF) [file pgen.1002945.s002.tif]

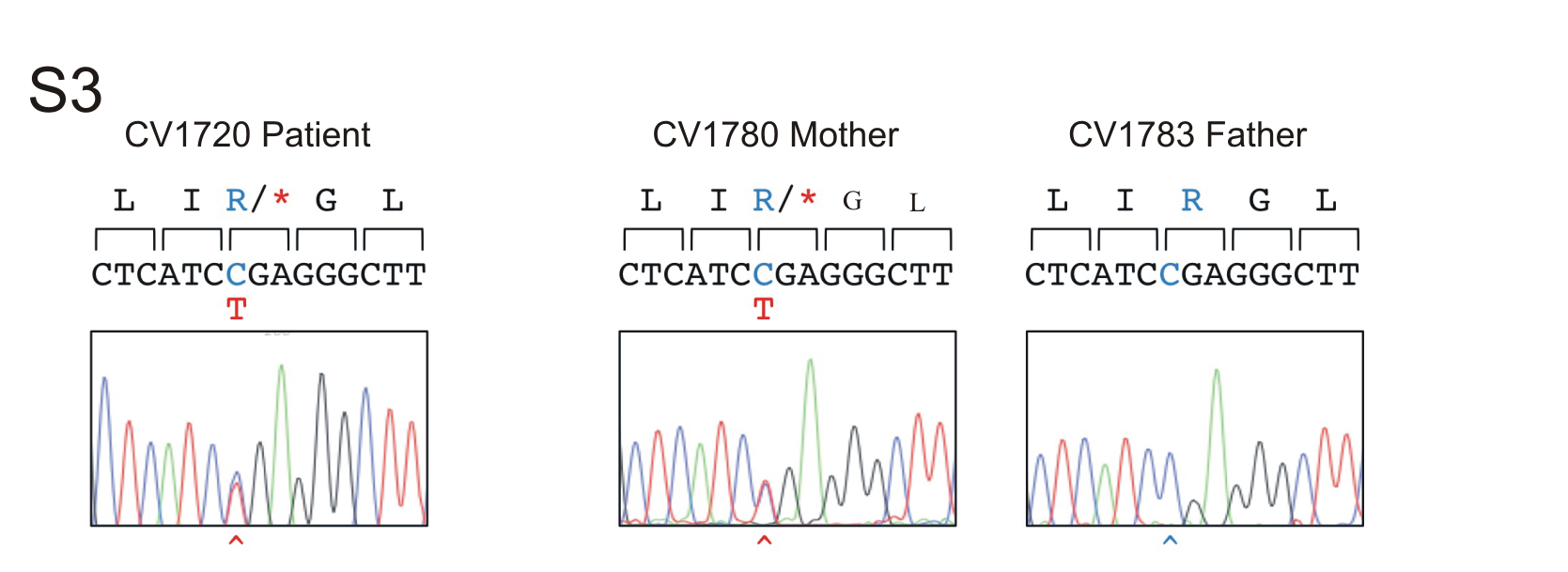

Supplement: Figure S3 — Identification of a truncating mutation in ATRIP in patient, CV1720. Genomic DNA sequencing of ATRIP exons showed that patient CV1720 and the unaffected mother, CV1780, are heterozygous for a c.2278C>T mutational change in exon12 of ATRIP. The father has a WT sequence at this site. 2278C>T generates a primary stop codon predicting a truncated protein at position arginine 760 (p.R760*). WT sequence shown in blue, the mutation is shown in Red. (TIF) [file pgen.1002945.s003.tif]

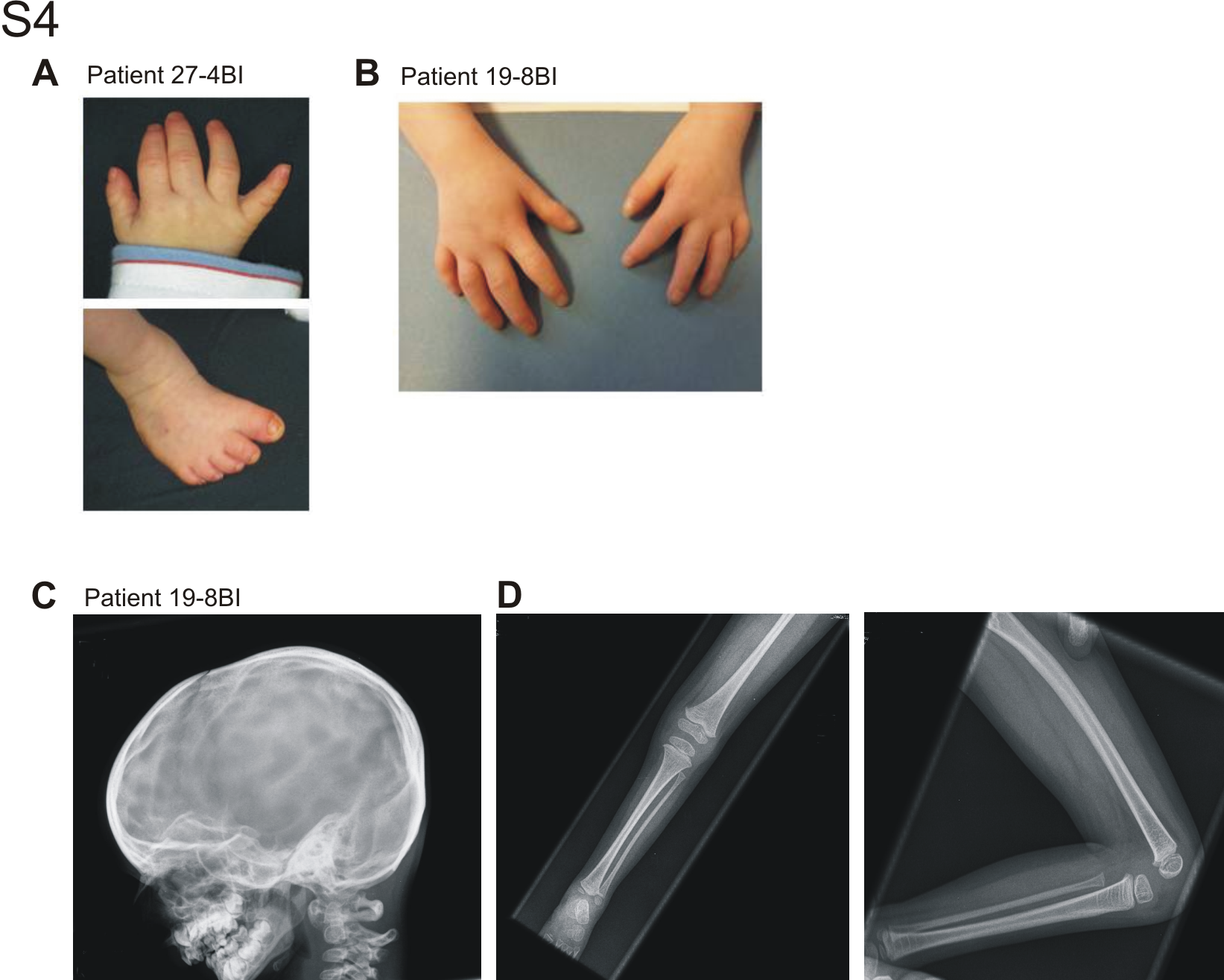

Supplement: Figure S4 — Photographs of patients 27-4BI and 19-8BI. A) Shows abnormal digits of patient 27-4BI. B) Copper beaten appearance of skull of patient 19-8BI. C) Frontal and Lateral view of left knee of patient 19-8BI showing an absence of ossification of the patella. (TIF) [file pgen.1002945.s004.tif]

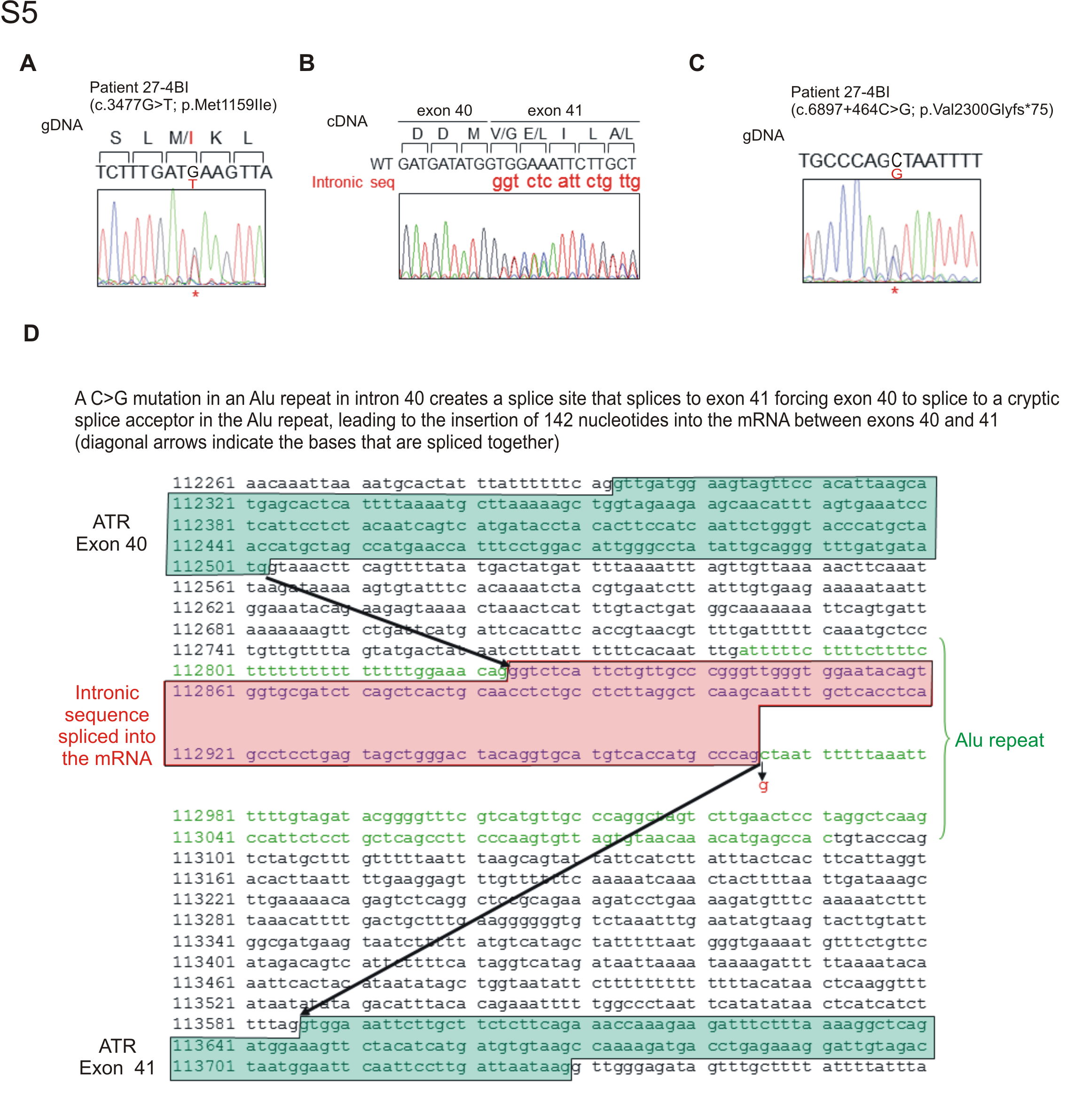

Supplement: Figure S5 — Mutational changes observed in ATR in patients 27-4BI and 19-8BI. A) c.3477G>T mutational change in patients 27-4BI and 19-8BI. RT-PCR sequencing revealed a heterozygous 3477G>T mutational change in both patients causing an amino acid substitution, p.Met1159Ile, which lies within a conserved UME (NUC010) domain of ATR. B) A double sequence was observed at the boundary between exons 40 and 41 in both patients. Sequencing showed that the double sequence was caused by insertion of a 142 bp region from intron 40. C) A C to G mutational change was observed in intron 41 of both patients converting the sequence CAGCT to CAGGT, a splice site. The insertion causes a frameshift and a stop codon at p.Val2300Glyfs*75. D) Diagram showing the likely origin of the insertion observed at the exon 40/41 boundary. Sequencing of intron 40 revealed a C>G mutation as indicated creating a cryptic splice site causing splicing of exon 40 to the indicated intronic sequence (which represents an Alu repeat sequence). Thus one ATR allele of the patients harbours a 142 nucleotide insertion between exons 40 and 41. Exons 40 and 41 are highlighted in green and the inserted intronic sequence is shown in red. The intronic C>G change is highlighted in red. The insertion causes a frameshift and a stop codon at c.6978 in exon 41. (TIF) [file pgen.1002945.s005.tif]
