# Supplementary material for: Identification of the First ATRIP–Deficient Patient and Novel Mutations in ATR Define a Clinical Spectrum for ATR–ATRIP Seckel Syndrome
Source: PLoS Genet. 2012 Nov 8;8(11):e1002945. doi: 10.1371/journal.pgen.1002945 (PMC3493446; doi:10.1371/journal.pgen.1002945)
Supplement: Table S1 — The table shows the position of single nucleotide polymorphisms identified in intron 1 and 2 in the patient and parental genomic DNA. * The contig position is defined as the position of the single nucleotide variant (SNV) on the contig (NT_022517.17) when counting from the first base (base position = 1). **rs# is the NCBI's reference SNP ID. *** minor allele (indicated as a base) and its frequency (MAF) (second most frequent allele) in a default global population reported in dbSNP database (1000 Genome phase 1, May 2011). N.A. not available. (DOCX) [file pgen.1002945.s006.docx]

Table S1 SNPs identified in *ATRIP* intron 1 and 2

|  | SNV alleles | | |  |  |  |
| --- | --- | --- | --- | --- | --- | --- |
| contig position^*^ | CV1720  (Patient) | CV1780  (Mother) | CV1783  (Father) | reference SNP cluster record^**^ | MAF^***^ | remarks |
| 48428196 | G/C | G/C | G | rs72622933 | C=0.119 | -59bp upstream of ATG start codon |
| 48429402 | A/G | G | A/G | rs922075 | A=0.447 | intron 1 |
| 48430197 | C | C | C | rs4858794 | G=0.068 | intron 1 |
| 48430612 | C/G | C | C/G | rs1824363 | G=0.296 | intron 1 |
| 48430845 | A/G | A | A/G | rs9311426 | N.A. | intron 1 |
| 48431354 | G/A | G/A | G/A | not reported | - | intron 1: -92bp upstream of exon 2 |
| 48431433 | A/G | A | A/G | not reported | - | intron 1: -13bp upstream of exon 2 |
|  |  |  |  |  |  |  |
|  |  |  |  |  |  |  |
